# Association of Apelin and Apelin Receptor Polymorphisms With the Risk of Comorbid Depression and Anxiety in Coronary Heart Disease Patients

**DOI:** 10.3389/fgene.2020.00893

**Published:** 2020-08-11

**Authors:** Ying Wang, Wenhui Liu, Yiwen Xiao, Haiyan Yuan, Feng Wang, Pei Jiang, Zhiying Luo

**Affiliations:** ^1^Department of Pharmacy, The Second Xiangya Hospital of Central South University, Changsha, China; ^2^Institute of Clinical Pharmacy, Central South University, Changsha, China; ^3^Institute of Clinical Pharmacy & Pharmacology, Jining First People’s Hospital, Jining Medical University, Jining, China

**Keywords:** coronary heart disease, depression, gene polymorphisms, apelin, anxiety

## Abstract

The Apelin (APLN)/apelin receptor (APLNR) signaling pathway is a newly identified regulator in various cardiovascular diseases, which is considered as a candidate pathway for the occurrence of coronary heart disease (CHD), depression, and anxiety. The goal of this study was to investigate the association between APLN/APLNR gene polymorphisms and the risk of depression and anxiety in CHD patients. To this end, a case-control study involving 269 CHD patients and 184 healthy control individuals was conducted. The 269 patients with CHD including 122 patients with and 147 patients without depression, and 56 patients with and 213 patients without anxiety Four single nucleotide polymorphisms were selected and successfully genotyped using Sanger sequencing. The APLN rs2235310T allele and APLNR rs9943582C allele were found to be associated with an increased risk of CHD after multiple test correction (*P*-adjust < 0.05). The patients with CHD who carried the rs9943582C allele had a higher risk of depression, after adjusting for alcohol drinking habits, insomnia, hypertension, and stroke history, with the Bonferroni correction (*P*-adjust = 0.018). The APLNR rs2282623 T allele was associated with an increased risk of anxiety in CHD patients after adjusting for related disease complications, with the Bonferroni correction (*P*-adjust = 0.022). We reported for the first time that the APLN rs2235310 and APLNR rs2282623 polymorphisms are associated with the risks of psychiatric disorders in CHD patients and may serve as novel biomarkers for therapy.

## Introduction

Coronary heart disease (CHD) is one of the most common chronic diseases, having a serious effect on human health and quality of life (Pezzella, 2010). Epidemiological studies show that CHD patients are more likely to suffer from psychiatric disorders, including depression and anxiety (Chauvet-Gélinier et al., 2013). Over the past few decades, an ever-expanding number of prospective reports and meta-analyses have provided evidence that depression and anxiety are risk factors for the morbidity and mortality in patients with and without established CHD (Lichtman et al., 2008; Carney and Freedland, 2017; Hohls et al., 2020). Patients with CHD, who also have depression or anxiety, have worse outcomes than do those without depression and anxiety (Hare et al., 2014). Researchers have suggested that the risks of mortality and cardiovascular events are directly correlated with the severity of depression, the more severe depression associated with more severe cardiac events (Rugulies, 2002). Meanwhile, cognitive-behavioral treatment and positive psychology intervention have been showed to improve several psychological symptoms and reduced cardiac mortality for patients with CHD or coronary artery disease (CAD; Richards et al., 2018; Magan et al., 2020). Hence, it’s quite important to disclose the relationship between CHD and psychical disorders.

Reasons for the relationship between CHD and psychical disorders are complicated, mainly including biological and behavioral mechanisms. An increasing number of studies show that CHD and psychiatric disorders share common biological mechanisms, including endothelial and platelet dysfunction, inflammation, autonomic dysfunction, and hypothalamus–pituitary–adrenocortical axis dysfunction (Martinez-Quintana et al., 2020). The behavioral factors, including poor adherence to medical treatment and physical inactivity, associate with increased risk of both CHD and depression (Kamphuis et al., 2007). In recent decade, numbers studies have suggested that genetic factors are important determinants of CHD and psychiatric disorders (Han et al., 2019).

The APLN/APLNR signaling pathway is involved in various pathological processes and physiological functions, including cardiovascular disease, angiogenesis, energy metabolism, and central nervous system disease (Lv et al., 2020). A previous meta-analysis has shown that the circulating apelin level is a prominent athero-protective marker against the development of CAD, while the APLNR rs9943582 polymorphism in the APLNR promoter, is associated with an increased risk of CAD (Chen et al., 2017). Furthermore, increased levels of serum apelin have been shown to be significant independent predictors of the development of depression and anxiety in patients on peritoneal dialysis (Gok Oguz et al., 2016).

Thus, the APLN/APLNR signaling pathway is considered a candidate pathway for the occurrence of CHD, depression, and anxiety. Therefore, the present study aimed to evaluate the association between APLN and APLNR gene polymorphisms and the risk of comorbid depression and anxiety in Chinese patients with CHD.

## Materials and Methods

### Subjects

A total of 269 patients with CHD and 184 matched healthy volunteers were enrolled in this study, which was conducted at the outpatient clinic of the Jining First People’s Hospital in Shandong Province and at the Second Xiangya Hospital in Hunan Province to investigate the effects of genetic polymorphisms in patients with CHD combination with depression and anxiety. This study was conducted in compliance with the stipulations of the Declaration of Helsinki, and the study protocol was approved by the Ethics Committee of the Jining First People’s Hospital. Written informed consent was received from each participant prior to the study, and the participants were identified by numbers throughout the study.

### Study Design

**FIGURE 1:**
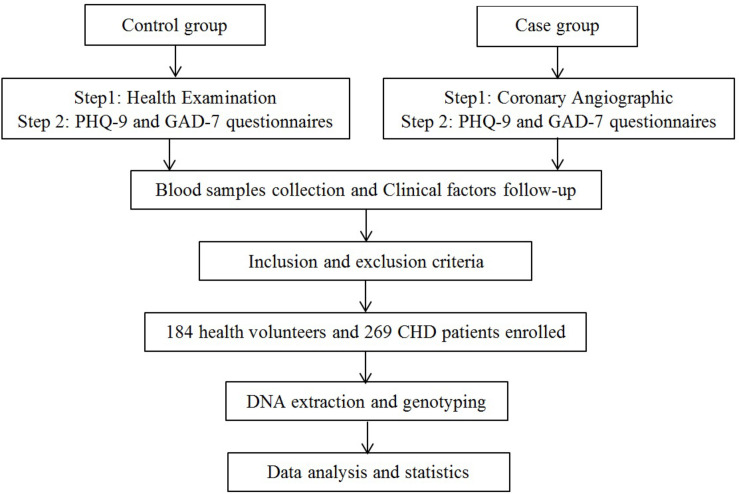
Study design.

The study design of this research is shown in Figure 1. The sample size for this research was calculated using Power and Sample Size Calculation version 3.1.2 (*Department of Biostatistics, Vanderbilt University, Nashville, TN, United States*) based on the following parameters: an independent case–control study; a type I error (α) of 0.05; a statistical power of 0.8; the probability of exposure in cases (p0) of 0.15; the probability of exposure in the control (p1) of 0.3; and the control to case ratio (m) of 0.8. Based on these parameters, the calculated minimum sample size was 137 experimental subjects and 110 control subjects.

### Diagnosis of CHD

The diagnosis of CHD was independently made by two experienced cardiologists based on the coronary angiographic findings in the patients. The reference standard for CHD diagnosis was the presence of severe coronary artery stenosis (more than 50%) in at least one major coronary artery or major branches. Patients were excluded if they had other serious disease including severe autoimmune disease, valvular heart disease, severe liver and/or kidney disease, and cancer.

### Symptoms of Depression and Anxiety

The presence of depression in the patients with CHD was evaluated by two experienced psychiatrists according to the criteria of the 5th Edition of the Diagnostic and Statistical Manual of Mental Disorders. The depression level was scored using the Patient Health Questionnaire-9 (PHQ-9), a nine-item questionnaire that is commonly used to screen for symptoms of depression in outpatients. To reduce the interference of environmental factors on questionnaire performance, all patients were required to complete the questionnaire alone in a separate room, unless they required assistance with writing or reading. We set a threshold of the PHQ-9 scale >5 points to indicate the possibility of depression.

The seven-item Generalized Anxiety Disorder 7 (GAD-7) scale, which is widely used to measure anxiety symptoms in the general population, was used to assess symptoms of anxiety in the patients. The process of evaluating anxiety is the same as evaluating depression. A score of five or greater on the GAD-7 scale represented a cutoff point for identifying symptoms of anxiety.

### Control Group

Healthy control individuals were selected among adults without CHD who had underwent several assessments including clinical physical examination, radiographic chest examination, electrocardiogram analysis, and evaluation of medical history. Moreover, all healthy volunteers completed questionnaires, including PHQ-9 and GAD-7 according to standard process. The presence of psychiatric disorders was evaluated by two independent psychiatrists based on the questionnaires. Finally, 184 age- and sex-matched healthy volunteers were enrolled in this study.

### Data Collection

Data were collected by reviewing the outpatient medical records. Clinical factors were mainly obtained from the electronic medical records, including demographic variables (e.g., age, sex, height, weight, and smoking and drinking habits) and coexisting diseases (e.g., hypertension, diabetes mellitus, and chronic gastritis).

### Single Nucleotide Polymorphism (SNP) Selection

We first systematically reviewed studies that reported genetic polymorphisms in the APLN/APLNR signaling pathway and selected previously investigated SNPs based on published studies found in PubMed. The SNP inclusion criteria were an association with a functional change, and a significantly associated with the risk of CHD and psychiatric disorders. The tag SNP strategy was further used to ensure a better detection rates, all the selected SNPs had a minor allele frequency >0.1 in Chinese or Asians. If several SNPs were in linkage disequilibrium (LD, *r*^2^ > 0.8), only one representative SNP was selected, e.g., rs2235309 was in a high LD with rs2235310, and rs7119375 was in a high LD with rs9943582. The final candidate tag SNPs were as follows: rs3115757 and rs2235310 in APLN, and rs9543582 and rs2282623 in APLNR.

### DNA Extraction and Genotyping

Peripheral venous blood (4 mL) was collected from each subject into an anticoagulation tube filled with sodium citrate. Genomic DNA was extracted using the TIANamp Blood DNA Kit (Tiangen, China) following the manufacturer’s instructions. Primers for PCR amplification and sequencing are listed in Supplementary Table S1. The PCR reaction was performed in a total volume of 25 μl reaction containing 2.5 μl of 10 × PCR buffer (TaKaRa Bio Inc., Japan), 2 μl dNTP mixture (TaKaRa Bio Inc.), 2 μl genomic DNA, 0.2 μl Taq polymerase (TaKaRa Bio Inc.), 0.5 μl of each primer and 17.3 μl of water. The PCR products were sequenced by Sanger sequencing, with the assisted of Shanghai Majorbio Bio-pharm Technology, Co., Ltd., Company.

### Statistical Analysis

All analyses were performed using SPSS Statistics version 19.0 (*SPSS, Inc., Chicago, IL, United States*). Data regarding the demographic, clinical, and genetic characteristics of the patients are presented as the mean ± standard deviation or counts (%) as appropriate. Differences in the demographic and clinical characteristics between the patients and healthy control individuals were evaluated using a *t*-test for continuous variables and χ^2^-test for categorical variables. The Hardy–Weinberg equilibrium (HWE) for polymorphisms was assessed using the χ^2^ test. Pairwise LD analyses were carried out using SHEsis. The genotype distributions and allele frequencies in the patients with CHD and controls were analyzed using the χ^2^ test. The Bonferroni adjustment was applied to correct for multiple comparisons. Differences in the risks of CHD and psychiatric disorders between groups were calculated using multivariate analysis of variance followed by the Bonferroni correction for multiple comparisons. *P*-values were adjusted for complications, when needed. Odds ratios and 95% confidence intervals were also calculated.

## Results

### Characteristics of the Study Participants

The demographic characteristics of the patients with CHD and healthy control individuals are provided in Table 1. No significant differences were observed between the patient and control groups (*P* > 0.05). The patients with CHD were further divided into the following subgroups, depending on whether comorbid depression (D) or anxiety (A) was present: CHD+D, CHD-D, CHD+A, and CHD-A. The demographic and clinical characteristics of the patients from these subgroups are shown in Table 2. Approximately 50% of the patients with CHD had depression or anxiety. Moreover, 51 of the 56 patients (91.7%) with anxiety had comorbid depression.

**TABLE 1:** Demographic characteristics of the enrolled subjects.

Variables	CHD (*N* = 269)	Control (*N* = 184)	*P*-value
Age (years)	61.24 ± 12.08	59.82 ± 11.79	0.39
Gender (M/F)	144/127	45/39	0.78
Smoking	87 (32.34%)	54 (29.35%)	0.54
Drinking	90 (33.46%)	46 (25.00%)	0.06
BMI	23.69 ± 1.98	23.47 ± 2.13	0.67

*CHD, coronary heart disease.*

**TABLE 2:** Demographic and clinical characteristics among different coronary heart disease (CHD) groups.

Variables	CHD (*N* = 269)	CHD+D (*N* = 122)	CHD-D (*N* = 147)	*P*_1_-value	CHD+A (*N* = 56)	CHD-A (*N* = 213)	*P*_2_-value
Age (years)	61.24 ± 12.08	61.62 ± 13.71	60.92 ± 10.60	0.64	61.00 ± 11.77	61.30 ± 12.18	0.87
Gender (M/F)	144/127	70/52	74/75	0.22	32/24	112/103	0.55
Smoking	87 (32.34%)	36 (29.51%)	51 (34.69)	0.43	16 (28.57%)	71 (33.33%)	0.53
Drinking	90 (33.46%)	35 (28.69%)	65 (44.22%)	**0.011**	17 (30.36%)	73 (34.27%)	0.64
BMI	23.69 ± 1.98	24.17 ± 2.05	23.86 ± 2.45	0.34	24.13 ± 2.48	24.85 ± 2.96	0.26
Insomnia	94 (34.94%)	58 (47.54%)	36 (24.49%)	**0.006**	28 (50.00%)	66 (30.98%)	**0.011**
Hypertension	208 (77.3%)	62 (50.82%)	146 (99.32%)	**6.77E-15**	32 (57.14%)	176 (82.63%)	**4.96E-62**
Diabetes mellitus	52 (19.33%)	22 (18.03%)	30 (20.41%)	0.645	9 (16.07%)	43 (20.19%)	**7.11E-57**
Stroke	32 (11.90%)	9 (7.38%)	23 (15.65%)	**0.039**	6 (10.71%)	26 (12.20%)	**3.18E-42**

*CHD+D, CHD patients with depression; CHD-D, CHD patients without depression; CHD+A, CHD patients with anxiety; CHD-A, CHD patients without anxiety. *P*_1_-value: CHD+D versus CHD-D; *P*_2_-value: CHD+A versus CHD-A. Bold values means: *p* < 0.05.*

The patients with CHD who had depression had a markedly lower rate of alcohol intake than did those without depression (28.69% vs. 44.22%, respectively; *P* = 0.011). Furthermore, the patients with comorbid depression or anxiety had an increased rate of insomnia relative to those without these psychiatric disorders (47.54% vs. 24.49% and 50.00% vs. 30.98%, respectively; *P* < 0.05). We also found that patients with CHD who had depression had markedly lower rates of comorbid hypertension and a stroke history than did those without depression (50.82% vs. 99.32% and 7.38% vs. 15.65%, respectively; *P* < 0.001). Similarly, the patients with anxiety symptoms had lower rates of comorbid hypertension, diabetes mellitus, and a stroke history than did those without anxiety symptoms (57.14% vs. 82.63%, 16.07% vs. 20.19%, and 10.71% vs. 12.20%, respectively; *P* < 0.001).

### Hardy–Weinberg Equilibrium Analysis

The following candidate tag SNPs were selected for analysis: rs3115757 and rs2235310 in APLN gene and rs9543582, and rs2282623 in APLNR gene. The genotypes of the four SNPs in the CHD and control groups were in HWE based on the χ^2^ test results, suggesting that the cases enrolled in this study were representative of the population as shown in Table 3. The LD analysis indicated that the SNPs studied were in a low LD with each other (*r*^2^ < 0.8), as shown in Supplementary Figure S1.

**TABLE 3:** Hardy–Weinberg equilibrium analysis of studied single nucleotide polymorphisms (SNPs) in coronary heart disease (CHD) patients and controls.

SNP-ID	Genotype	CHD			Control		
			
		Number	X_1_^2^	*P*_1_	Number	X_2_^2^	*P*_2_
APLN: rs3115757	GG GC CC	134 107 25	0.29	0.59	83 76 15	0.17	0.68
APLN: rs2235310	TT TC CC	167 79 14	1.29	0.26	115 59 9	0.16	0.69
APLNR: rs9943582	TT TC CC	122 107 38	1.37	0.24	94 73 12	2.18	0.14
APLNR: rs2282623	CC CT TT	121 112 35	3.08	0.079	89 73 18	0.28	0.59

*APLNR, apelin receptor.*

### Association Between Gene Polymorphisms and CHD Risk

The frequency distributions of the genotypes and alleles of the four selected SNPs between the patients with CHD and controls are shown in Table 4. The data showed that the C allele of the APLN rs2235310 polymorphism occurred less frequently in the former group (19.96% vs. 30.53%, respectively; *P* = 2.38E-4) and, thus, might be a protective factor against the development of CHD. After the Bonferroni correction, the *P*-value for the rs2235310 polymorphism and the risk of CHD was less than 0.01. By contrast, the CC genotype and C allele of the APLNR rs9943582 polymorphism were associated with an increased risk of CHD (*P* = 0.04 and *P* = 0.027, respectively). And the APLNR rs9943582 C allele was still significantly associated with the incidence of CHD after multiple test correction (*P* = 0.014). However, no significant relationships between the other two SNPs and the risk of CHD were observed.

**TABLE 4:** Association between *APLNR/APLNR* gene mutations and patients with CHD.

Gene	SNP-ID	CHD (*N* = 269)	Control (*N* = 184)	*P*-value	*P*-adjust
APLN	rs3115757 GG GC CC	134 (50.38%) 107 (40.22%) 25 (9.39%)	83 (47.70%) 76 (43.68%) 15 (8.62)	0.77	1.00
	G C	375 (70.67%) 157 (29.33%)	242 (69.54%) 106 (30.46%)	0.76	1.00
	rs2235310 TT CT CC	167 (64.23%) 79 (30.38%) 14 (5.39)	115 (62.84%) 59 (31.24%) 9 (5.12%)	0.91	1.00
	T C	413 (80.04%) 103 (19.96%)	289 (69.47%) 127 (30.53%)	**2.38E-4**	**<0.01**
APLNR	rs9943582 TT TC CC	122 (45.69%) 107 (40.07%) 38 (14.34%)	94 (52.51%) 73 (40.78%) 12 (6.71%)	**0.040**	0.06
	T C	351 (65.73%) 183 (34.26%)	261 (72.90%) 97 (27.10%)	**0.027**	**0.014**
	rs2282623 CC CT TT	121 (45.15%) 112 (41.79%) 35 (13.06%)	89 (49.44%) 73 (40.56%) 18 (10.00%)	0.52	0.32
	C T	374 (69.78%) 188 (30.22%)	251 (69.72%) 109 (30.28%)	0.35	0.21

*P-adjust means P-value adjusted with Bonferroni test. APLNR, apelin receptor; CHD, coronary heart disease; SNP, Single nucleotide polymorphism. Bold values means: *p* < 0.05.*

### Influence of Gene Polymorphisms on the Risk of Depression in CHD Patients

**FIGURE 2:**
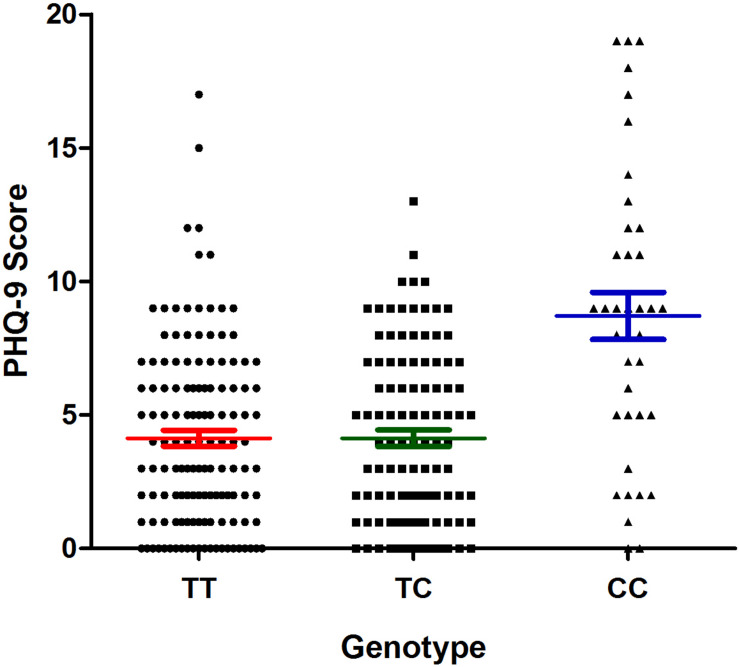
Association of apelin receptor (APLNR) rs9943582 polymorphism with PHQ-9 scores in coronary heart disease (CHD) patients with comorbid depression. The filled circle means TT genotype carriers, the filled square means TC genotype carriers and the filled triangle means the CC genotype carriers. The red lines mean the mean ± SD of PHQ-9 score of TT group, the green lines mean the mean ± SD of PHQ-9 score of the TC group, and the blue lines mean the mean ± SD of the PHQ-9 score of the CC group.

The results of the association analysis between the selected polymorphisms and the risk of depression in patients with CHD are shown in Table 5. We found that only the rs9943582 data reached statistical significance at the *P* < 0.05 level. Both the CC genotype and C allele were associated with a higher risk of depression in patients with CHD (21.00% vs. 8.78% and 40.76% vs. 29.06%; *P* = 0.013 and *P* = 0.006, respectively). Furthermore, the association between rs9943582 and the risk of depression remained significant after adjusting for alcohol drinking habits, insomnia, hypertension, and the stroke history, with the Bonferroni correction (*P*-adjust = 0.042 and *P*-adjust = 0.018, respectively). We also examined the association of rs9943582 with the severity of depressive symptoms and found that the PHQ-9 scores were markedly higher in the patients with CC genotype than in those with the other genotypes (*P* < 0.001; Figure 2).

**TABLE 5:** Genotype distribution of polymorphisms between CHD+D and CHD-D.

SNP	CHD+D (*N* = 121)	CHD-D (*N* = 148)	*P*-value	*P*-adjust*
rs3115757 GG GC CC	61 (51.69%) 45 (38.13%) 12 (10.28%)	73 (49.32%) 62 (41.89) 13 (8.79%)	0.80	1.00
G C	167 (70.76%) 69 (29.24%)	208 (70.27%) 88 (29.73%)	0.92	1.00
rs2235310 TT CT CC	78 (68.42%) 31 (27.19%) 5 (4.39%)	89 (60.96%) 48 (32.88%) 9 (6.16%)	0.45	0.62
T C	187 (82.02%) 41 (17.98%)	226 (77.40%) 66 (22.60%)	0.23	0.58
rs9943582 TT TC CC	47 (39.50%) 47 (39.50%) 25 (21.00%)	75 (50.68%) 60 (40.54%) 13 (8.78%)	**0.013**	**0.042**
T C	141 (59.24%) 97 (40.76%)	210 (70.94%) 86 (29.06%)	**0.006**	**0.018**
rs2282623 CC CT TT	61 (50.83%) 43 35.83) 16 (13.16%)	63 (43.45%) 63 (43.45%) 19 (13.10%)	0.42	0.74
C T	165 (68.75%) 75 (31.25%)	189 (65.17%) 101 (34.83%)	0.40	0.98

*P-value adjustment for drinking habit, insomnia, hypertension, and stroke history, multiple comparisons with Bonferroni test. CHD, coronary heart disease. Bold values means: *p* < 0.05.

### Influence of Gene Polymorphisms on the Risk of Anxiety in CHD Patients

**FIGURE 3:**
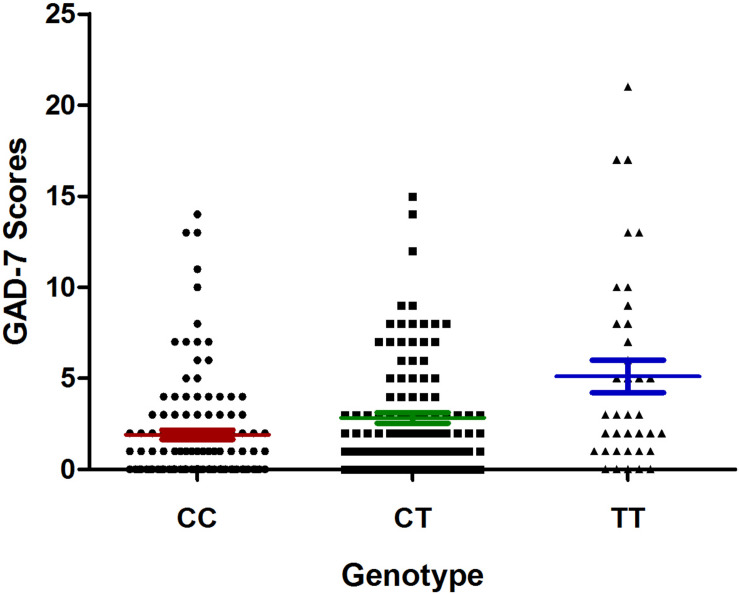
Association of apelin receptor (APLNR) rs2282623 polymorphism with generalized anxiety disorder 7 (GAD-7) scores in coronary heart disease (CHD) patients with comorbid anxiety. The filled circle means CC genotype carriers, the filled square means CT genotype carriers and the filled triangle means the TT genotype carriers. The red lines mean the mean ± SD of GAD-7 score of CC group, the green lines mean the mean ± SD of GAD-7 score of the CT group, and the blue lines mean the mean ± SD of the GAD-7 score of the TT group.

The results of the association analysis between the studied mutations and the risk of anxiety are shown in Table 6. The C allele of the rs9943582 polymorphism was associated with a lower risk of anxiety in patients with CHD (25.46% vs. 36.73%, respectively; *P* = 0.032). However, the association did not remain statistically significant after adjusting for insomnia, hypertension, diabetes mellitus, and the stroke history, with the Bonferroni correction (*P*-adjust = 0.081). The TT genotype and T allele of the APLNR rs2282623 polymorphism were associated with a higher risk of anxiety in patients with CHD (19.64% vs. 11.32% and 47.32% vs. 30.42%; *P* = 0.003 and *P* = 0.001, respectively). These associations remained statistically significant after adjusting for complications (*P*-adjust = 0.017 and *P*-adjust = 0.022, respectively). The rs2282623 polymorphism was also associated with the severity symptoms of anxiety, with the GAD-7 scores being higher in the CC genotype carriers than in the TT + TC genotype carriers (*P* = 0.013; Figure 3).

**TABLE 6:** Genotype distribution of polymorphisms between CHD+A and CHD-A.

SNP	CHD+A (*N* = 56)	CHD-A (*N* = 213)	*P*-value	*P*-adjust*
rs3115757 GG GC CC G C	28 (50.0%) 23 (41.07%) 5 (8.93%) 79 (70.54%) 33 (29.46%)	106 (50.48%) 84 (40.09%) 20 (9.52%) 296 (70.48%) 124 (29.52%)	0.98 1.00	0.95 1.00
rs2235310 TT CT CC T C	33 (58.93%) 20 (35.71%) 3 (5.36%) 86 (76.78%) 26 (23.22%)	134 (65.69%) 59 (28.92%) 11 (5.39%) 327 (80.15%) 81 (19.85%)	0.61 0.43	0.53 0.47
rs9943582 TT TC CC T C	32 (57.14%) 20 (35.71%) 4 (7.15%) 82 (74.54%) 28 (25.46%)	90 (42.65%) 87 (41.23%) 34 (16.12%) 267 (63.27%) 155 (36.73%)	0.088 **0.032**	0.24 0.11
rs2282623 CC CT TT C T	14 (25.00%) 31 (55.36%) 11 (19.64%) 59 (52.68%) 53 (47.32%)	107 (50.47%) 81 (38.21%) 24 (11.32%) 295 (69.58%) 129 (30.42%)	**0.003** **0.001**	**0.017** **0.022**

*P-value adjustment for drinking habit, insomnia, hypertension, and stroke history, multiple comparisons with Bonferroni test. Bold values means: *p* < 0.05.

## Discussion

In this study, we investigated the associations of four promising polymorphisms in the APLN/APLNR pathway with the risks of depression and anxiety in patients with CHD. Our principal findings demonstrated that genetic polymorphisms in the APLN/APLNR pathway might result in a potential risk for depression and anxiety in patients with CHD. To the best of our knowledge, this is the first pilot study exploring the genetic contribution of the APLN/APLNR pathway to the susceptibility to depression and anxiety in Chinese patients with CHD.

We first found that patients with CHD who experienced insomnia had a high risk of comorbid depression and anxiety, which was consistent with previous findings, demonstrating that insomnia is frequently co-morbid with depression and anxiety (Gebara et al., 2018). Additionally, patients with CHD who has alcohol drinking habits were less likely to have depression than were non-drinkers. This finding, however, deviates from previously published results, which suggested a causal relationship between alcohol use disorders and major depression (Agabio et al., 2018). Moreover, patients with CHD who had more complications were more likely to have depression or anxiety, which may be explained by the additional psychological stress that these patients experience.

Apelin is an endogenous peptide capable of binding to the apelin receptor. Both apelin and its receptor are widely distributed in various tissues, including the central nervous and cardiovascular systems, and play a dominant role in cardiovascular homeostasis and disease (Chen et al., 2015). The beneficial effects of the apelin/apelin receptor pathway are well established. Apelin treatment has been proven beneficial for conditions as diverse as hypertension, atherosclerosis, myocardial infarction, and other cardiovascular diseases (Zhou et al., 2016). Numerous studies have shown that APLN/APLNR polymorphisms are associated with the risk of several diseases such as hypertension, CAD, and diabetes mellitus (Nowzari et al., 2018; Wu et al., 2018; Zhang et al., 2019). Our results demonstrated that the T allele of the rs2235310 polymorphism and the C allele of the rs9943582 polymorphism were risk factors in the development of CHD.

The association between rs9943582, a functional variant in the 5’ flanking region (-154G/A) of APLNR, and the incidence of cardiovascular disease has been extensively studied; however, the results have been contradictory. One study showed that the rs9943582 polymorphism is associated with the expression levels of APLNR. In particular, C allele carriers had lower expression level of the APLNR because of a lower binding affinity of a transcription factor to the promoter of APLNR (Hata et al., 2007). A published meta-analysis showed that the T allele of rs9943582 is associated with an increased risk of CAD (*P* = 0.100) relative to that of the wild-type C allele (Chen et al., 2017). Additionally, a previous genome-wide association study conducted in a Japanese population, showed a significant association between rs9943582 and the risk of stroke (Hata et al., 2007), whereas no allelic or genotypic associations were found between rs9943582 and ischemic stroke in a Chinese Han population (Wang et al., 2017; Zhang et al., 2017). The APLN rs2234306 polymorphism was in a strong LD with the APLN rs3115757 polymorphism in the Chinese population from this study (*r*^2^ > 0.9), and both SNPs were located in APLN introns. Previous studies showed that the rs3115757 polymorphism is associated with the expression level of APLN (Liao et al., 2011) and the incidence of diabetes and hypertension (Huang et al., 2016; Zheng et al., 2016). Our findings support a moderate contribution of the APLN/APLNR pathway polymorphisms to the development of CHD in Chinese patients.

The APLN/APLNR pathway may also serve as a promising therapeutic target for the treatment of psychosis and neuropathy (Lv et al., 2020). We further investigated the influence of APLN/APLNR pathway polymorphisms on the risks of depression and anxiety in patients with CHD. Among the patients with CHD, the rs9943582 C-allele carriers had an increased risk of depression, whereas rs2282623 T-allele carriers had a higher risk of anxiety. This is the first pharmacogenomics study to examine the associations between polymorphisms located in genes of the APLN/APLNR pathway and the susceptibility to depression and anxiety in patients with CHD.

Apelin-13 has exhibited antidepressant and anxiolytic effects in different animal models (Telegdy and Jaszberenyi, 2014; Dai et al., 2018). A functional study of the rs2282623 SNP, located in the 3′UTR of APLNR, showed that the rs2282623 T-allele is associated with the decreased levels of apelin-13 and nitrite (Mishra et al., 2015). Moreover, another study demonstrated that the rs2282623 mutation is associated with diastolic blood pressure and the mean arterial pressure response to a low-sodium intervention (Zhao et al., 2010). In the current study, we showed that the SNPs associated with low apelin expression levels were also associated with an increased risk of depression or anxiety in patients with CHD.

However, other studies have shown controversial roles of APLN in psychosis. For example, patients on peritoneal dialysis, who had depression and anxiety, had higher serum apelin levels than those without depression and anxiety (Gok Oguz et al., 2016). Furthermore, apelin-13 exhibited depression-promoting effects in both forced swimming and tail suspension tests (Lv et al., 2012). Hence, additional pharmacogenomics studies with a larger sample size should be conducted to further validate the results of this study.

This study has several limitations. First, the candidate SNP approach was used to identify emotional disorder associated polymorphisms in patients with CHD. This approach might have missed mutations that are truly associated with the studied phenotypes. Second, although the final sample size was larger than the minimum calculated sample size, the results of this study should be verified using a larger sample size. More large-sample-size, high-quality, multicenter clinical trials are urgently needed to validate the association of gene polymorphisms in the APLN/APLNR pathway on the risk of emotional disorders in patients with CHD.

## Conclusion

The present study supports the hypothesis that APLN/APLNR polymorphisms contribute to the susceptibility to depression and anxiety in Chinese patients with CHD. Replication studies with larger samples are required to verify the role of these polymorphisms in patients with CHD, who comorbid depression and anxiety.

## Data Availability Statement

The raw data supporting the conclusions of this article will be made available by the authors, without undue reservation.

## Ethics Statement

The studies involving human participants were reviewed and approved by the Jining First People’s Hospital. The patients/participants provided their written informed consent to participate in this study.

## Author Contributions

ZL and PJ designed the experiments and drafted the manuscript. YW and WL accomplished works of the data collection, DNA extraction, and genotyping. YX and HY helped to enroll the all of the patients. FW guided to conduct the statistic works. All authors contributed to the article and approved the submitted version.

## Conflict of Interest

The authors declare that the research was conducted in the absence of any commercial or financial relationships that could be construed as a potential conflict of interest.
