# Supplementary material for: Association of Apelin and Apelin Receptor Polymorphisms With the Risk of Comorbid Depression and Anxiety in Coronary Heart Disease Patients
Source: Front Genet. 2020 Aug 11;11:893. doi: 10.3389/fgene.2020.00893 (PMC7432257; doi:10.3389/fgene.2020.00893)
Supplement: Supplementary file 1 [file Data_Sheet_1.docx]

**APLNR**

**APLN**


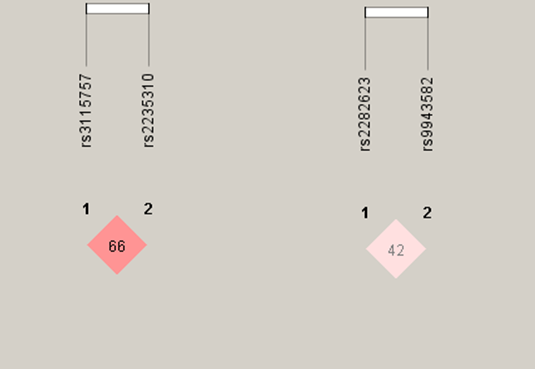


Figure S1. The LD analysis of SNP located in APLN/APLNR pathway.

Table S1. The PCR primers for tagSNPs in this study.

| SNPs | Primers | Product size | sequencing primer |
| --- | --- | --- | --- |
| rs3115757 | F 5’-TGCCCGTTTCCAACAACCTTCC-3’  R 5’-CTCCGCTTTAGCACTGTCCACTG-3’ | 262 | F-primer |
| rs2235310 | F 5’-CCTCTGCTTCCCTTGCCTTAGTTC-3’  R 5’-CCTCCACTGCCTCCTGACCTG-3’ | 291 | F-primer |
| rs9543582 | F 5’-TCCCTCCTCCCACCTCCAGAC-3’  R 5’-TGCCCATCCTGCGAAATCTTACAC-3’ | 400 | F-primer |
| rs2282623 | F 5’-TTCCCCTCTCCTCCTCCCTCTG-3’  R 5’-CCTCTTGGCTTCTGCTGGATTGG-3’ | 290 | F-primer |

Note: F, forward primer; R, reverse primer.
